# Epidemiological analysis of varicella in Dalian from 2009 to 2019 and application of three kinds of model in prediction prevalence of varicella

**DOI:** 10.1186/s12889-022-12898-3

**Published:** 2022-04-07

**Authors:** Tingting Cheng, Yu Bai, Xianzhi Sun, Yuchen Ji, Fan Zhang, Xiaofeng Li

**Affiliations:** 1 Department of Epidemiology and Health Statistics, Dalian Medical University, 9 Lvshun South Road, Dalian, 116044 People’s Republic of China; 2 Dalian Center for Disease Control and Prevention, 78 Taiyuan Street, Dalian, 116021 People’s Republic of China

**Keywords:** Varicella, Epidemic characteristics, GM (1,1) model, Markov model GM (1,1)-Markov model

## Abstract

**Objective:**

This study described the epidemic characteristics of varicella in Dalian from 2009 to 2019, explored the fitting effect of Grey model first-order one variable( GM(1,1)), Markov model, and GM(1,1)-Markov model on varicella data, and found the best fitting method for this type of data, to better predict the incidence trend.

**Methods:**

For this Cross-sectional study, this article was completed in 2020, and the data collection is up to 2019. Due to the global epidemic, the infectious disease data of Dalian in 2020 itself does not conform to the normal changes of varicella and is not included. The epidemiological characteristics of varicella from 2009 to 2019 were analyzed by epidemiological descriptive methods. Using the varicella prevalence data from 2009 to 2018, predicted 2019 and compared with actual value. First made GM (1,1) prediction and Markov prediction. Then according to the relative error of the GM (1,1), made GM (1,1)-Markov prediction.

**Results:**

This study collected 37,223 cases from China Information System for Disease Control and Prevention's “Disease Prevention and Control Information System” and the cumulative population was 73,618,235 from 2009 to 2019. The average annual prevalence was 50.56/100000. Varicella occurred all year round, it had a bimodal distribution. The number of cases had two peaks from April to June and November to January of the following year. The ratio of males to females was 1.17:1. The 4 to 25 accounted for 60.36% of the total population. The age of varicella appeared to shift backward. Students, kindergarten children, scattered children accounted for about 64% of all cases. The GM(1,1) model prediction result of 2019 would be 53.64, the relative error would be 14.42%, the Markov prediction result would be 56.21, the relative error would be 10.33%, and the Gray(1,1)-Markov prediction result would be 59.51. The relative error would be 5.06%.

**Conclusions:**

Varicella data had its unique development characteristics. The accuracy of GM (1,1)—Markov model is higher than GM(1.1) model and Markov model. The model can be used for prediction and decision guidance.

**Supplementary Information:**

The online version contains supplementary material available at 10.1186/s12889-022-12898-3.

## Background

Varicella is an acute infectious disease caused by the first infection with varicella-zoster virus (VZV). The incubation period is about 2 weeks, and some cases can reach 3 weeks. It is highly contagious from the day before the rash to full scab healing. The main transmission route is respiratory droplets or direct contact infection. Varicella patients are the only source of infection [1, 2].

Varicella has a high infection rate in China. Varicella usually heals on its own. Individuals, infants, and adults with low immune function may be serious [3]. VZV is a teratogen and causes congenital varicella syndrome (CVS) [4]_._ Moreover, VZV is a common cause of stroke [5]. Varicella has complicated complications and causing serious economic burden. Vaccines are the most economical method [6].

The prevalence of varicella in China ranks first among vaccine-preventable infectious diseases [7]. Studies found that children were vaccinated with varicella vaccine, the protective effect of the vaccine declined year by year, varicella would break out again [8]. Some severe breakthrough varicella can occur, but it does not seem to be common [9]. Research shows, the incidence of varicella has shown an overall upward trend [10, 11]. Adults infected varicella easier and more dangerous. In many places, the age of varicella had been found to shift backward [12]. Studies have found that adults in tropical and subtropical countries were more likely to suffer from varicella than in countries with mild climates [13]. Many factors like air pressure, temperature, humidity, rainfall, and other subtle factors can have a complex impact on the incidence of varicella [14]. There are differences in different regions, and the incidence of varicella is different. Find a model suitable for the varicella data in Dalian, explore the fitting effects of different models on the data, and provide a theoretical basis for varicella prediction and health decision-making.

The gray model was proposed by Professor Julong Deng in 1982 [15]. It is a widely used data prediction model. It can suit various data types. It seeks valuable information by fully extracting and developing small samples and poor information data. And it generates predictions to demonstrate the direction of the system. Grey model first-order one variable (GM (1,1))is the core of the gray model and it was used widely. While in varicella prediction, the GM (1,1) was less used.

The Markov chain theory was proposed by a famous mathematics scientist A.A. Markov in 1906, The Markov model was maturely used in the economic field and played an important role in medical dynamics prediction [16], Markov prediction means that in the process of data transfer, under the action of a certain factor, their state probability depends on the previous result, and the probability law of the Nth result depends only on the result of the (N-1)th experiment. And has nothing to do with the earlier results, the process is a random process [17], Predict the state of the process at the next moment and the next few moments through the law of change between the states of the random process at different moments [18].

The general experience of the Grey forecast dynamic model believes that its prediction accuracy for random, long-term, and volatile historical data is low, and it is mainly suitable for reflecting the overall development trend of the forecast [19]. While the Markov forecast is mainly based on the current state and the law of state transition predicts the possible state of the system in the future, generally through the transition probability matrix for prediction and decision-making. It can just make up for the limitation of the gray model. Therefore, try to use the Markov principle to fix the gray prediction value. Combine the advantages of two models to establish a Gray(1,1)-Markov prediction model [20].

In this paper, two parts were included. First, an epidemiological description of varicella in Dalian from 2009 to 2019. Second, GM (1,1) model, Markov model, and GM(1,1)-Markov model were established to fit the incidence of varicella and to explore the fitting effects of different models on varicella data. It can better find the development trend of varicella and the change law of epidemic cycle, guide the formulation of medical policy, reasonably allocate medical resources and avoid the waste of medical resources. It can also evaluate the effect of vaccine, isolation and other prevention and control measures, so as to provide a scientific theoretical basis for the prediction of disease outbreak, the selection of effective prevention and control measures in advance, and the prevention of varicella outbreak.

## Materials and methods

### Materials

The data in this study was collected from Dalian Centers for Disease Control and Prevention (CDC). The varicella data from 2009 to 2019 includes the total infection number, age distribution, months distribution, and the occupation distribution. Among them, the occupation distribution in 2009 was missed. The GM (1, 1) model and Markov model was built with data from 2009 to 2018.The GM (1, 1)-Markov model was built according to the result of the GM (1, 1) model.

### Descriptive epidemiological analysis

The epidemiological characteristics of varicella in Dalian from 2009 to 2019 was described by time and population. Statistical charts were adopted to intuitively describe the epidemic characteristic.

### GM (1,1) model

The data used from 2009 to 2018 as the original data, established a GM (1, 1) model and predicted the number of varicella in 2019, and analyzed the accuracy of the GM (1,1) model prediction results.

### Markov model

The data used from 2009 to 2018 as the original data. K-mean cluster was adopted to divide groups. The state transition matrix was used to predict the 2019 group matrix and Markov model prediction value.

### GM (1,1)-Markov model

The relative value between the predicted value of the GM (1,1) model from 2009 to 2018 and the true value was used as the original data. K-mean cluster was used to divide groups. The state transition probability matrix was calculated according to grouping. 2018, 2017, and 2016 were selected as the three most recent years from 2019. The three-step transition probability matrix was used to calculate the state interval of 2019. GM (1,1) model prediction value was combined to calculate GM (1,1)-Markov model prediction value.

### Statistic software

The epidemiological analysis of varicella data was collected and analyzed by Excel 2010 software. R software 3.2.5 version was adopted to conducted GM (1,1) model prediction, the posterior difference ratio (C), and the probability of small error (P) were used to evaluate the effect of prediction of the GM (1,1) model. Markov model and GM (1,1)-Markov model was implemented with the help of MATLAB 2015a software. Relative error was selected as the index to evaluated the accuracy of the models.

## Result

### Epidemic characteristics

#### Time distribution

**Fig. 1:**
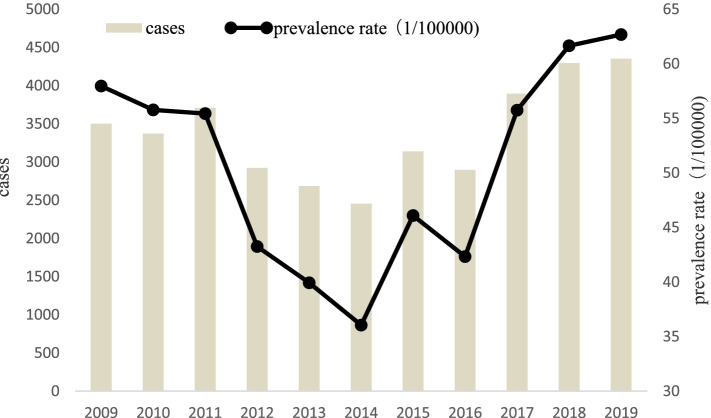
The cases and pervalencce rate of varicella in Dalian from 2009 to 2019

**Fig. 2:**
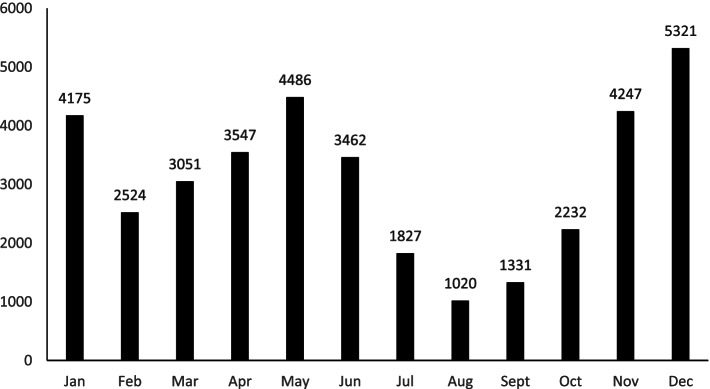
Month distribution of varicella cases in Dalian from 2009 to 2019

The total number of reported cases of varicella in Dalian from 2009 to 2019 was 37,223. The prevalence rate decreased and then increased on the whole. The lowest prevalence rate was occurred in 2014 (see Table 1, Fig. 1). Varicella occurred throughout the year. The number of cases was increasing from February to May and August to December. From April to June, and November to January of the following year, there were more cases (see Fig. 2). Varicella cases showed obvious seasonality.

**Table 1:** The cases and prevalence rate of varicella in Dalian from 2009 to 2019

Year	Cases	Demographic data	Prevalence rate(1/100000)
2009	3502	6,042,554	57.96
2010	3371	6,043,668	55.78
2011	3709	6,690,429	55.44
2012	2922	6,756,401	43.25
2013	2684	6,721,652	39.93
2014	2455	6,809,204	36.05
2015	3139	6,811,207	46.09
2016	2897	6,843,225	42.33
2017	3895	6,986,442	55.75
2018	4296	6,968,856	61.65
2019	4353	6,944,597	62.68

### Population distribution

#### Age distribution

**Fig. 3:**
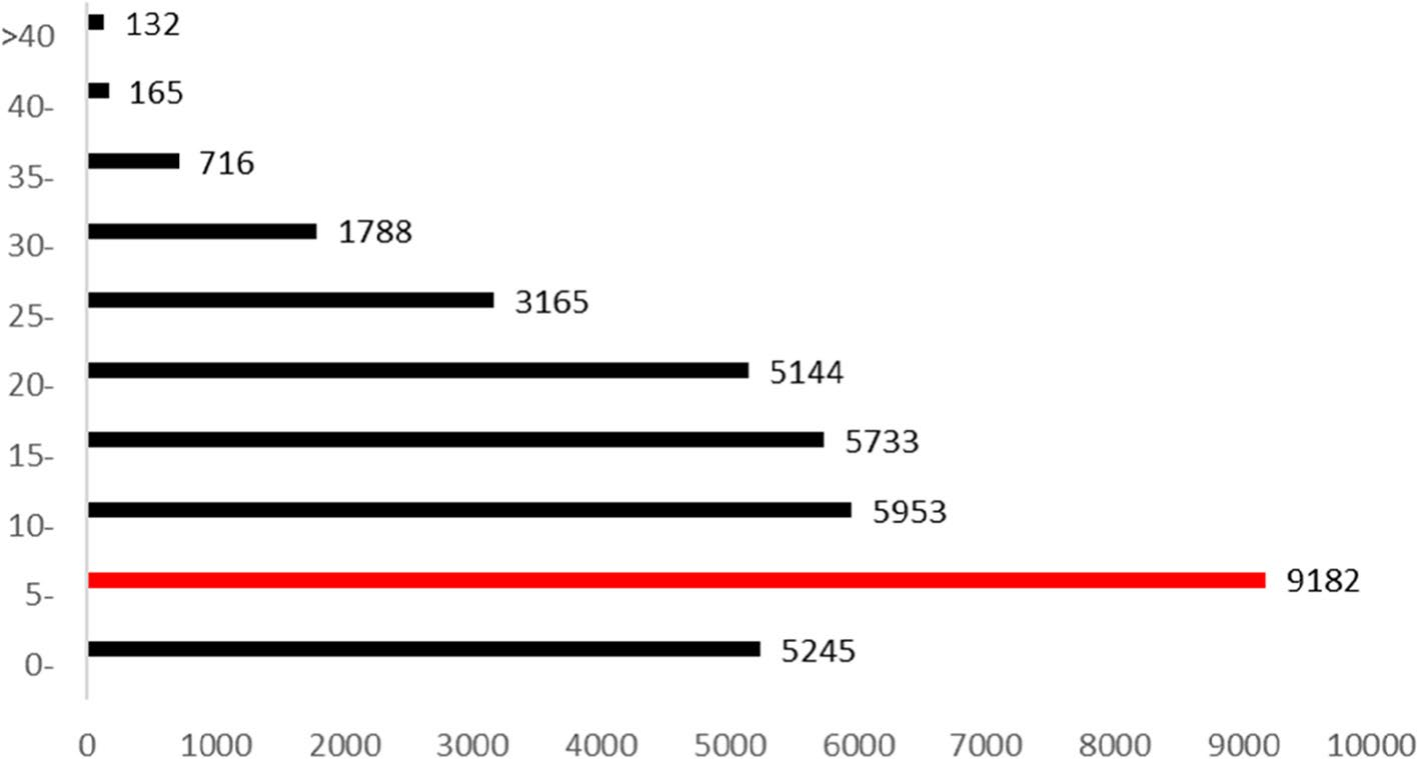
The varicella cases age distribution in Dalian from 2009 to 2019

Varicella occurred any at age. The cases aged from 0 to 25 accounted for 92.46% of the total cases. More cases aged from 5 to 10 (see Fig. 3).

#### Gender distribution

**Fig. 4:**
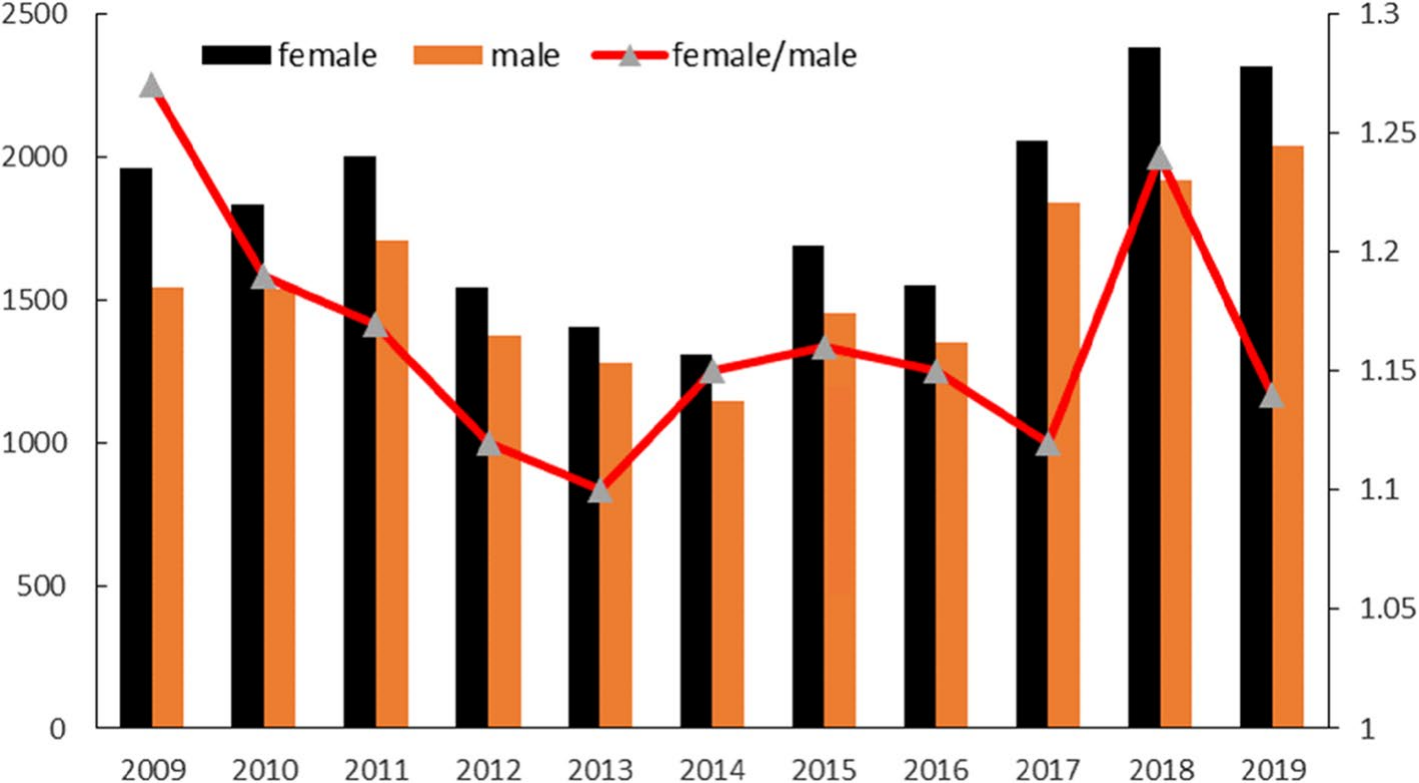
Total cases and gender distribution of varicella in Dalian from 2009 to 2019

The total cases of varicella in Dalian from 2009 to 2019 was 37,223, of which 20,043 cases were males (53.85%) and 17,180 cases were females (46.15%). The number of males was higher than that of females, and the ratio of total male to female was 1.17:1. (Fig. 4).

#### GM (1,1) model

Using R software for GM (1,1) model prediction. The prevalence for 2019 was 51.30/100000 (Table 2), the posterior difference ratio (C) was 0.99, the probability of small error (P) was 0.33.α = -0.01,u = 45.38. Then the GM (1, 1) model was established as follows:

**Table 2:** GM (1,1) model prediction results with R software

Year	_Actual value (1/100000)_	R software prediction value(1/100000)
2009	57.96	57.96
2010	55.78	46.30
2011	55.44	46.83
2012	43.25	47.37
2013	39.93	47.91
2014	36.05	48.46
2015	46.09	49.01
2016	42.33	49.58
2017	55.75	50.15
2018	61.65	50.72
2019	62.68	51.3O

$${x}^{\left(1\right)}\left(k+1\right)=4038.28{e}^{\left(0.52k\right)}-3980.32$$

### Markov model

#### State division and state transition probability matrix

Due to the amount of data was small, to ensure that each state had enough data, it was divided into 3 states, K-mean cluster was used to cluster data. The data distribution of each group could be determined. State division was: E1 [36.05, 41.13], E2 [41.13, 50.77], E3 [50.77, 61.65].

The state change from 2009 to 2018 is showed as E3-E3-E3-E2-E1-E1-E2-E2-E3-E3, state transition status and state transition probability matrix see Tables 3 and 4. State transition matrix showed below:

**Table 3:** State transition from 2009 to 2018

State	E1	E2	E3
E1	1	1	0
E2	1	1	0
E3	0	1	3

**Table 4:** State transition matrix

State	E1	E2	E3
E1	0.50	0.50	0
E2	0.33	0.33	0.33
E3	0	0.25	0.75

$$P=\left(\begin{array}{ccc}0. 5& 0. 5& 0\\ 0. 33& 0. 33& 0. 33\\ 0& 0. 25& 0. 75\end{array}\right)$$

#### Use the state transition probability matrix to predict

With the help of MATLAB 2015a software, using the 2018 grouping matrix (0, 0, 1) and the state transition probability matrix to predict. The result of the 2019 grouping matrix was (0, 0.25, 0.75), the 2019 Markov model prediction was more likely in E3, so the value is: 56.21.

### GM (1,1)-Markov model

#### State division and state transition probability matrix

Relative value was used to divide the state, relative value = actual value/ R software prediction value. K-mean cluster was used to cluster data. The data distribution of each group could be determined. Divided all relative values into three states: underestimated, accurate, overestimated, namely E1 [1.10,1.22], E2 [0.82,1.10], E3[0.74,0.82].The status changed from 2009 to 2018 is E2-E1-E1-E2-E2- E3-E2-E2-E1-E1 (Table 5). State transition matrix see Table 6.

**Table 5:** State transition from 2009 to 2018

Year	Actual value	Predicted value	Relative value	State division
2009	57.96	57.96	1	E2
2010	55.78	46.30	1.20	E1
2011	55.44	46.83	1.18	E1
2012	43.25	47.37	0.91	E2
2013	39.93	47.91	0.83	E2
2014	36.05	48.46	0.74	E3
2015	46.09	49.01	0.94	E2
2016	42.33	49.58	0.85	E2
2017	55.75	50.15	1.11	E1
2018	61.65	50.72	1.22	E1
2019	62.68	51.30

**Table 6:** State transition matrix

State	E1	E2	E3
E1	0.67	0.33	0
E2	0.40	0.40	0.20
E3	0	1	0

#### Three-step transition probability matrix to prediction varicella in 2019

Select 2018, 2017, and 2016, the three most recent years from 2019. After one step (P1), two steps (P2), and three steps (P3), the state was transferred to 2019. Sum the column items, the maximum value was the state range, which the 2019 GM(1,1) model predicted value located in, see Table 7.

**Table 7:** Status of the predicted values of the GM(1,1)model in 2019

Year	Initial state	transition steps	E1	E2	E3
2018	E1	1 (P1)	0.67	0.33	0
2017	E1	2 (P1)	0.58	0.35	0.07
2016	E2	3 (P3)	0.48	0.42	0.10
sum			1.73	1.10	0.17

$$P\left(1\right)=\left(\begin{array}{ccc}0. 67& 0.33& 0\\ 0. 4& 0. 4& 0. 2\\ 0& 1& 0\end{array}\right) P\left(2\right)=P{\left(1\right)}^{2}=\left(\begin{array}{ccc}0. 5809& 0. 3531& 0. 066\\ 0. 428& 0. 492& 0. 08\\ 0. 4& 0. 4& 0. 2\end{array}\right) P\left(3\right)=P{\left(1\right)}^{3}\left(\begin{array}{ccc}0. 5304& 0. 3989& 0. 0706\\ 0. 4836& 0. 418& 0. 0984\\ 0. 4& 0. 4& 0. 2\end{array}\right)$$

The maximum sum value was in state E1, That was the GM(1,1) model prediction value will be in the E1 state, the prediction value of 51.30 was been underestimated, and the GM (1,1)-Markov prediction value was 51.30*(1.10 + 1.22)/2= 59.51.

### Models results comparison

**Fig. 5:**
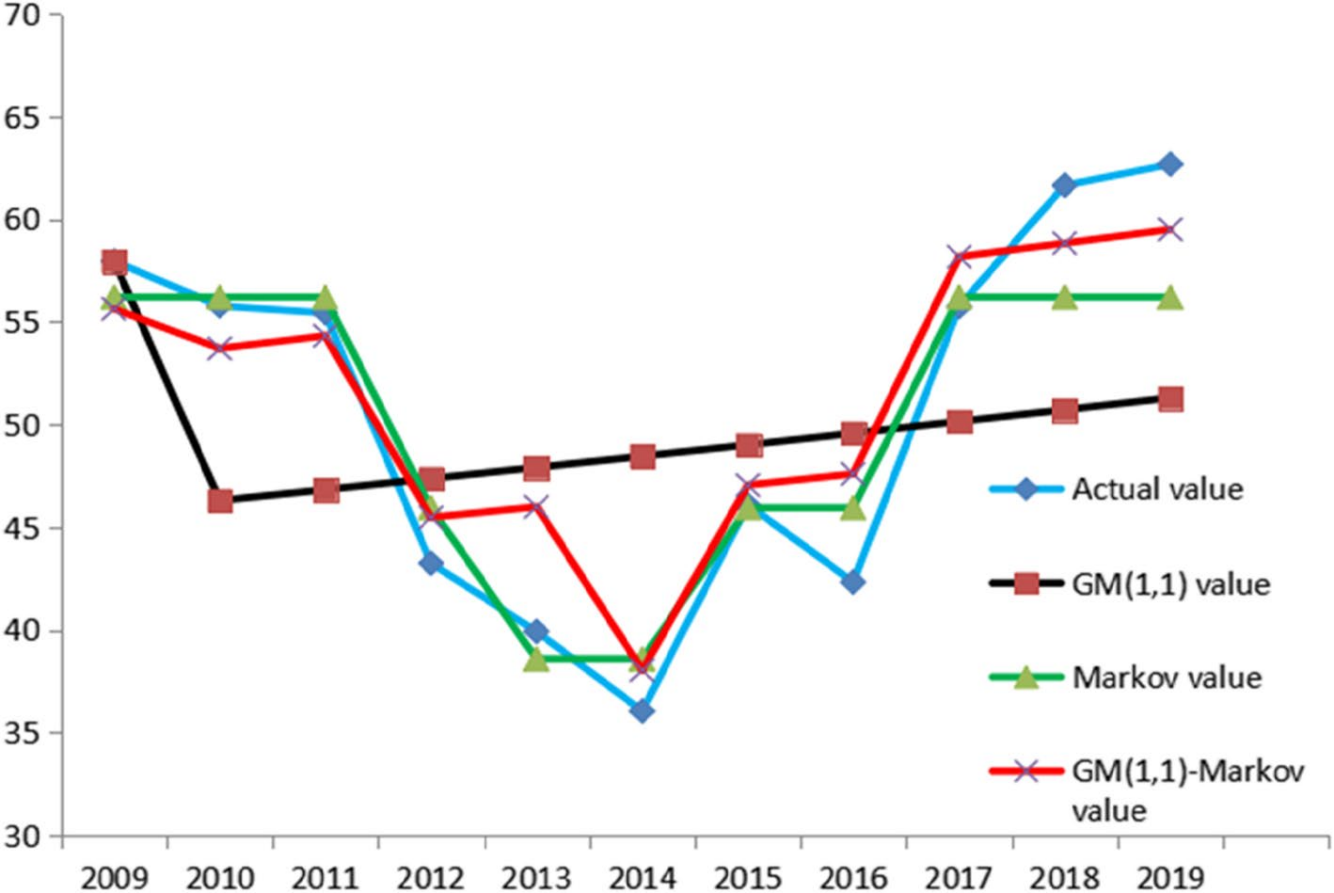
Comparison between prediction value and actual value

Three models results and comparison see Fig. 5 and Table 8. GM (1,1)-Markov model fitted actual value better and had the lowest relative error of 2019.

**Table 8:** Models results and relative errors of 2019

Year	Actual value	GM (1,1)	Markov	GM (1,1)-Markov
2019	62.68	53.64	56.21	59.51
Relative error(%)		14.42	10.31	5.06

## Discussion

There were 37,223 varicella cases and the cumulative population was 73,618,235 in Dalian from 2009 to 2019. The average annual prevalence was 50.56/100000. Although varicella patients have obtained lifelong immunity and no longer becomes an exposed population, it has not been removed from the total population because it accounts for a small proportion of the total population. The high prevalence was corresponding with our country's overall situation [7]. Varicella cases showed an upward trend just like other places [10, 11]. Varicella can occur all year round, mostly in spring and winter, seasonality is obvious. The two peaks of varicella cases were from April to June and November to January of the following year, showing a "bimodal distribution". This time the temperature change violently, people especially children who had low immunity prone to be infected. This time for children also studied at school. So, they can contact the outside world and other infected people easier. The effective way to prevent chickenpox is vaccination, but because chickenpox vaccine is a class II epidemic Vaccine, not all parents are willing to be vaccinated against varicella. And varicella outbreaks still occur. The prevention and control of varicella epidemic still needs to be strictly grasped. Considering the two peak periods and incubation periods of varicella, it is suggested that Dalian take the 13th and 44th weeks of each year as the early warning week of varicella, and the key objects of prevention and control are kindergarten children and primary and secondary school students. Before the school season begins, the community health service centers should further guide health care and health education for kindergartens and schools with high prevalence of varicella, so as to effectively reduce the prevalence rate of varicella. Each street (town) community health service center shall strengthen the daily supervision of kindergartens and primary and secondary schools, strictly carry out morning and afternoon inspection, early detection, early isolation of patients, implement the disinfection of the place of onset, carry out health publicity and health education for key populations, improve the vaccination awareness of varicella vaccine and improve the vaccination rate of the second dose of varicella vaccine [21, 22].

In this paper, students, childcare, scattered children are the main disease groups. Most cases age from 5 to 10. The cases aged from 0 to 25 accounted for 92.46% of the total cases. High-age cases account for a large proportion. In many places, the age of varicella had shifted backward [12, 23, 24]. Adults infected varicella became easy and common. Adult varicella is more harmful. Adults especially during pregnancy get more symptoms of varicella and more dangerous [25–27]. Many reasons like a vaccine, weather, climate, and others have a complication effect on the incidence of varicella [8, 13, 14]. We still need to find out the reason and solution to decrease the incidence.

The prevalence ratio of varicella between men and women is 1.17:1, and the number of cases in men is higher than that in women. In many places, the varicella prevalence rate, the male is higher than that of the female. And there is a statistical difference [12, 27, 28]. Student, childcare, scattered children accounted for 73.15% of the total of the cases. So varicella outbreaks were prone to occur in schools, kindergartens. These places require to Strengthen prevention and control.

The GM (1,1), Markov model, and Gray-Markov were used to predict the prevalence of varicella in 2019. The predicted values were: 53.64, 56.21, and 59.51, and the relative errors of the true value in 2019 were: 14.42,10.33, and 5.06. From the results, the GM (1,1) model predicted the varicella cases were not ideal, and the model fitted poorly. Consider two follow reasons: 1The sample size was too small. 2 The sample size was too large. 3 The data was volatile. Concerning Tingting Zhang, Yanling Peng, and other documents [29], they collected 8 years of data from 2011 to 2018 and conducted GM (1,1) model prediction. The prediction level is excellent, and the amount of data is small, which is not the main reason for the unsatisfactory prediction. Besides, the GM (1,1) model is famous for predicting "small samples". There are also articles showing that the GM(1,1) model can be developed with only 4 models [30], and the sample size was small, which was not the main problem. Research showed that fewer and recent modeling data have a good forecast effect [31]. When the number of modeling data between 4 and 10. When the dimension was 4, the prediction accuracy was the highest. With the increase of dimension, the precision did not increase [32]. But in this study, the number of GM (1,1) modeling data should be the same as Markov modeling data. More data, especially much old data had a bad influence on GM (1,1) model. The accuracy of the Grey model was inevitably decreased. The GM(1,1) model had a good predictive effect for sequences with short sequences and an upward trend [33]. The prediction results of the GM(1,1) model were generally smooth curves, reflecting long-term growth trends [34], Poor fit effect for fluctuating data. Therefore, considering the large fluctuation of the sample was another reason for the unsatisfactory GM(1,1) prediction of this experiment. Consider further processing to improve forecast accuracy with this kind of data type. In this study, we combined GM(1,1) model and the Markov model to improve accuracy.

Markov predicted value in 2019 was 56.21, which was different from the actual value of 62.68. The status division had a greater impact. Theoretically speaking, the longer the historical data, the more state division, the higher the prediction accuracy [35]. Generally, when there was less historical data, the number of states should be less, so that each state had more data. When there were more data, more sample falls into each state. The more the state increased, the more accurate the obtained prediction interval would be. In this study, we had less data, less state, and the transition change situation could not be better reflected. The state interval span was large, and the data prediction could not be effective and accurate. Besides, the state division based on the original data, the Markov model could not express the trend of continuous growth. Therefore, Markov prediction error would become larger and larger.

The Grey-Markov prediction result was better than the Grey model and Markov model prediction alone. The Gray model was widely used in predicting small sample data. It could describe the overall trend well, but it could not reflect data fluctuations well. The Markov model had a good predictive effect on fluctuating data. However, the data was less and the state was insufficient, which easily led to poor prediction results. And it could not describe monotonic change data well. Grey-Markov model forecasting can combine the advantages of the GM(1,1) model to predict small samples and the advantage of Markov's response to fluctuations, improving the prediction accuracy to a certain degree. GM(1,1)-Markov forecast achieved a relatively good forecast result with the small sample, volatility, and overall risen data. In further studies, GM(1,1)-Markov model can be used in similar medical data to predict disease development and formulate reasonable medical health measures. And we still need to continuously optimize the model and explore better ways to get better results.

## Conclusion

The prevalence of varicella fluctuated greatly and showed an upward trend as a whole, and the age of onset shifted backward. GM (1,1)-Markov model can combine the advantages of small samples predicted by GM (1,1) model and the advantages of Markov model response fluctuation to improve the prediction accuracy. Varicella data was small and volatility, and good prediction results were obtained by using GM (1,1)-Markov model. GM (1,1)-Markov model can be used, improved and popularized in the prediction of similar types of data in the future.

## Supplementary Information

**Additional file 1.**

## Data Availability

The data that support the findings of this study are available from Dalian Centers for Disease Control and Prevention (CDC) but restrictions apply to the availability of these data, which were used under license for the current study, and so are not publicly available. Data are however available from the authors upon reasonable request and with permission of Dalian Centers for Disease Control and Prevention (CDC).

## Abbreviations

P: The probability of small error
C: The posterior difference ratio
CDC: Centers for Disease Control and Prevention
GM (1,1): Grey model first-order one variable
VZV: Varicella-zoster virus
