# Supplementary material for: Epidemiological analysis of varicella in Dalian from 2009 to 2019 and application of three kinds of model in prediction prevalence of varicella
Source: BMC Public Health. 2022 Apr 7;22:678. doi: 10.1186/s12889-022-12898-3 (PMC8991558; doi:10.1186/s12889-022-12898-3)
Supplement: Supplementary file 1 — Additional file 1. [file 12889_2022_12898_MOESM1_ESM.docx]

**Abbreviations**

P=the probability of small error, C= the posterior difference ratio CDC= Centers for Disease Control and Prevention, GM (1,1)= grey model first-order one variable, VZV= varicella-zoster virus

Declarations

Ethics approval and consent to participate

Not application

Consent for publication

Not application

Acknowledgements

Not application

Availability of data and materials

The data that support the findings of this study are available from Dalian Centers for Disease Control and Prevention (CDC) but restrictions apply to the availability of these data, which were used under license for the current study, and so are not publicly available. Data are however available from the authors upon reasonable request and with permission of Dalian Centers for Disease Control and Prevention (CDC).

Competing interests

The authors declare that they have no competing interests.

Funding

Not application

Authors’ contributions

Author1.CT:Conducted the research, did data entry，processed and analysis the data，and was a major contributor in writing the manuscript.

Author2.BY: Provided the original data.

All authors read and approved the final manescript.

Acknowledgements

Not application
